# Geriatrische Komplexbehandlung bei alterstraumatologischen Patienten: Einfluss auf Mobilität, Selbsthilfefähigkeit und Austrittsdestination

**DOI:** 10.1007/s00391-020-01812-4

**Published:** 2020-11-17

**Authors:** M. Palzer, U. Meyer, L. A. Abderhalden, A. Gazzotti, C. Hierholzer, H. A. Bischoff-Ferrari, G. Freystätter

**Affiliations:** 1 Zentrum Alter und Mobilität, Universität Zürich, Zürich, Schweiz; 2 Klinik für Geriatrie, Universitätsspital Zürich, Rämistrasse 101, 8091 Zürich, Schweiz; 3 Klinik für Traumatologie, Universitätsspital Zürich, Zürich, Schweiz; 4 Universitäre Klinik für Akutgeriatrie, Stadtspital Waid, Zürich, Schweiz

**Keywords:** Geriatrische frührehabilitative Komplexbehandlung, Sturz, Alterstraumatologie, Mobilität, Selbsthilfefähigkeit, Acute geriatric treatment, Fall, Orthogeriatrics, Mobility, Activities of daily living

## Abstract

**Hintergrund:**

Die geriatrische frührehabilitative Komplexbehandlung (GFK) wird bei hochbetagten hospitalisierten Patienten eingesetzt, um die Selbstversorgungsfähigkeit wiederherzustellen und eine Pflegebedürftigkeit zu vermeiden.

**Ziel der Arbeit:**

Ziel der Arbeit war es, die Veränderungen von Mobilität und Selbsthilfefähigkeit bei alterstraumatologischen Patienten* im Rahmen der GFK zu beschreiben.

**Material und Methoden:**

Mobilität, Ganggeschwindigkeit und Selbsthilfefähigkeit von 164 hospitalisierten Alterstraumatologiepatienten wurde zu Beginn und bei Abschluss der GFK erfasst. Wir analysierten die Veränderungen der Mobilität während GFK (t-Test), und welche Mobilitätsmerkmale mit einer Entlassung nach Hause vs. einer Entlassung in die Langzeitpflege assoziiert sind (alters- und geschlechtsadjustiertes Regressionsmodell).

**Ergebnisse:**

Die Patienten verbesserten ihre Mobilität gemessen mittels Short Physical Performance Battery (SPPB) um 1,8 ± 2,1 Punkte, die Ganggeschwindigkeit um 0,10 ± 0,14 m/s und den Barthel-Index um 13 ± 16 Punkte (alle *p* < 0,001). Die Zahl nichtgehfähiger Patienten verringerte sich von 43 auf 14 % (*p* = 0,003). Die Mehrzahl (73 %) der vor der Hospitalisation zu Hause lebenden Patienten wurde direkt oder nach einer überbrückenden spitalexternen Rehabilitation nach Hause entlassen.

**Schlussfolgerung:**

Die Datenanalyse zeigt signifikante und klinisch relevante Verbesserungen in den Bereichen Mobilität und Selbstständigkeit bei Alterstraumatologiepatienten. Die Mehrzahl der Patienten konnte wieder nach Hause austreten.

**Zusatzmaterial online:**

Zusätzliche Informationen sind in der Online-Version dieses Artikels (10.1007/s00391-020-01812-4) enthalten.

## Abstract (en)

**Background:**

Acute geriatric treatment is a type of early rehabilitation for hospitalized seniors to maintain personal autonomy and to avoid nursing home placement.

**Objective:**

The aim of the study was to describe the changes of mobility and functional independence of older trauma patients during acute geriatric treatment.

**Material and methods:**

This study analyzed admission and discharge assessment data from 164 patients in the geriatric department with fall-related injuries. Mobility and performance in activities of daily living were assessed using the short physical performance battery (SPPB), gait speed and Barthel index. We analyzed changes in mobility from admission to discharge (t-test) and examined differences in mobility between patients returning home and those admitted to long-term care (age-adjusted and gender-adjusted linear regression model).

**Results:**

Patients improved their mobility measured by the SPPB by 1.8 points ± 2.1 points, gait speed by 0.10 ± 0.14 m/s and the Barthel index by 13 ± 16 points, all *p* < 0.001). The number of patients not able to walk decreased from 43% to 14% (*p* = 0.003). Of the community-dwelling patients 73% were discharged either directly back home or after rehabilitation outside the hospital as a transitional solution.

**Conclusion:**

In the context of acute geriatric treatment older trauma patients significantly improved their mobility and performance. The majority of patients could return home.

## Hintergrund und Fragestellung

Im hohen Alter mobil zu bleiben, trägt entscheidend zur Unabhängigkeit von Senioren bei. Ein Verlust der Mobilität ist mit einem erhöhten Mortalitätsrisiko, einer verminderten Lebensqualität und einem erhöhten Risiko für Stürze assoziiert [1, 2]. Stürze und sturzbedingte Verletzungen sind im Alter häufig: Jeder dritte Senior über 65 Jahre und jeder zweite über 80-Jährige erleidet mindestens einen Sturz pro Jahr, rund 10 % der Stürze verursachen eine Verletzung [3]. Wirbelkörper‑, Becken- und Hüftfrakturen sind mit Immobilisation und erhöhtem Risiko für eine dauerhafte Einschränkung der Mobilität und Autonomieverlust assoziiert [4]. Das Ziel der geriatrischen Akutbehandlung ist neben der Behandlung der akuten Erkrankung die Wiederherstellung der Mobilität und der Selbstständigkeit im Alltag [5].

Frakturen beim älteren Patient sind häufig mit geriatrischen Syndromen wie Malnutrition, Delir und Mobilitätsverlust assoziiert. Zur optimalen Versorgung der Patienten hat sich über die letzten Jahre die Alterstraumatologie mit einem interdisziplinären und interprofessionellen Team etabliert [6]. Prestmo et al. zeigte 2015 in einer randomisierten kontrollierten Studie, dass sich die Mobilität von älteren Patienten mit Hüftfraktur unter geriatrischer Akutbehandlung deutlicher als unter rein orthopädischer Behandlung verbesserte [7]. Das orthogeriatrische Komanagement stellt heute den „standard of care“ bei der Behandlung alterstraumatologischer Patienten dar und ist in Deutschland für Patienten mit hüftgelenknahen Femurfraktur und positivem geriatrischen Screening eine Mindestanforderung. Die Datenlage abseits der hüftnahen Frakturen hinsichtlich Wiederherstellung der Selbstständigkeit und Rückkehr nach Hause im Rahmen der GFK bei alterstraumatologischen Patienten ist unbefriedigend [6].

Ziel der vorliegenden Studie war es, die Auswirkungen der GFK auf Mobilität, Selbstständigkeit und Aufenthaltsort nach Spitalentlassung bei älteren Patienten mit einer sturzbedingten Verletzung zu erfassen.

## Studiendesign und Untersuchungsmethoden

### Studiendesign und Patientenkollektiv

Die geriatrischen Assessments aller alterstraumatologischen Patienten, die von Januar 2016 bis Juli 2017 eine GFK in der Klinik für Geriatrie am Universitätsspital Zürich durchführten, wurden analysiert. Als weitere Einschlusskriterien mussten die schriftliche Patientenzustimmung zur Weiterverwendung gesundheitlicher Daten und ein Assessment mit einem Mobilitätstest zu Beginn und Abschluss der GFK vorliegen. Die Ethik-Kommission des Kantons Zürich bewilligte die Durchführung dieser Studie (2017-01573). Diese Studie ist Teil des Zürich-Forschungsschwerpunkts „peri-operative care in senior trauma patients“ (POPS) am Zentrum Alter und Mobilität der Universität Zürich und der Klinik für Geriatrie am Universitätsspital Zürich.

### Geriatrische frührehabilitative Komplexbehandlung

Nach der Erstversorgung ihrer sturzbedingten Verletzung wurden die Patienten für die GFK in die Klinik für Geriatrie verlegt. Die GFK beinhaltete: (1) ein standardisiertes geriatrisches Assessment zu Beginn der Behandlung und vor Entlassung, (2) die Behandlung durch ein geriatrisches Team unter altersmedizinisch-fachärztlicher Leitung, Physio- und Ergotherapie, Ernährungsberatung, mindestens 10 Trainingseinheiten/Woche, aktivierende, zur Selbstständigkeit befähigende Pflege durch speziell geschultes Pflegepersonal sowie (3) wöchentliche Teambesprechungen unter Beteiligung aller Berufsgruppen.

### Geriatrisches Assessment

Folgende Tests des geriatrischen Assessments wurden für diese Studie verwendet:

Die *Short Physical Performance Battery (SPPB)* ist ein validiertes Tool zur Beurteilung des Funktionsstatus der unteren Extremitäten [8]. Sie besteht aus einem Gleichgewichtstest, einem „Repeated-sit-to-stand“-Test und einem 4‑m-Gehtest [9]. Es ergibt sich ein Score von 0 bis 12 Punkten, wobei höhere Werte eine bessere Funktion darstellen.

Die *Ganggeschwindigkeit* ist ein einfach zu messender und guter Prädiktor für Verlust an Selbstständigkeit [10]. Wir ermittelten die Ganggeschwindigkeit (in Metern/Sekunde) mittels 4‑m-Gehtest im Rahmen der SPPB.

Zur Beurteilung des funktionellen Status wurde der *Barthel-Index* erfasst [11]. Daraus wird ein Score von 0 bis 100 Punkten gebildet, wobei 100 Punkte einer kompletten Selbstständigkeit entsprechen[11]. Mittels *Mini Nutritional Assessment (MNA)* klassifizierten wir den Ernährungszustand in normaler Ernährungszustand (24 bis 30 Punkte), Risiko für eine Mangelernährung (17 bis 23,5 Punkte) und Mangelernährung (unter 17 Punkte) [12] und mittels *Mini Mental Status Test (MMST)* nach Folstein et al. [13] die Kognition (normale Kognition 24 bis 30 Punkte, milde Kognitionsstörung 19 bis 23 Punkte, moderate Kognitionsstörung 10 bis 18 Punkte, schwere Kognitionsstörung <10 Punkte).

Körpergröße (cm) und -gewicht (kg) wurden im Assessmentcenter mittels Metermaß und geeichter Stehwaage von einer „assessment nurse“ ermittelt und daraus der Body-Mass-Index (BMI) errechnet. Aufenthaltsort nach Spitalentlassung, Hauptdiagnose sowie die Anzahl der Komorbiditäten (Nebendiagnosen) wurden dem klinischen Informationssystem entnommen. Die Spitalentlassung erfolgte in eine der folgenden Destinationen: nach Hause, in eine Rehabilitationsklinik, in eine stationäre Akut- und Übergangspflege oder in ein Alters- oder Pflegeheim. Bei der stationären Akut- und Übergangspflege werden intensive pflegerisch-therapeutische Leistungen erbracht.

### Datenanalyse

Die deskriptive Statistik zur Beschreibung der geschlechtsspezifischen Patientencharakteristika wurde mit χ^2^-Tests für kategorische und Student’s *t*-Tests für kontinuierliche Variablen durchgeführt. Die Veränderung der Mobilität wurde mit einem gepaarten Student’s *t*-Test geprüft. Zur Überprüfung möglicher Einflussfaktoren wurde zudem ein lineares Regressionsmodell verwendet, welches für Geschlecht (Männer vs. Frauen), Alter, Dauer des Spitalaufenthalts sowie die Anzahl der Komorbiditäten (0–5 vs. 6–10 vs. >10 Nebendiagnosen) adjustiert wurde. Da nicht alle Patienten in der Lage waren, zu Beginn den Gehtest durchzuführen, wurde die Ganggeschwindigkeit zudem als binäre Variable (in der Lage zu gehen vs. nicht in der Lage zu gehen) mittels McNemar’s Test ausgewertet.

Zur Auswertung des Ortes der medizinischen Anschlussbehandlung wurden die Kategorien „zurück nach Hause“ und „in eine Rehabilitationsklinik“ zusammengeführt und verglichen mit einer kombinierten Kategorie „ins Alters‑/Pflegeheim“ und „in eine stationäre Akut- und Übergangspflege“. Die Mobilitätswerte der 2 Kategorien wurden mittels multivariablem linearem „repeated-measures model“ miteinander verglichen. Dafür wurden die Indikatoren Zeitpunkt (Beginn, Ende), Entlassungsdestination (nach Hause/Reha, ins Heim/Übergangspflege) und deren Interaktion geprüft. Zudem wurde das Modell für Alter und Geschlecht adjustiert. In einem weiteren Schritt berechneten wir den prognostischen Wert der unterschiedlichen Mobilität-Scores zu Beginn und der Differenz zwischen Beginn und Ende auf die Austrittsdestination mittels einer für Alter und Geschlecht adjustierten logistischen Regression und überprüften, ob die Anzahl der Komorbiditäten oder der Traumatyp (Hüft‑/Beckenringfraktur vs. Schädel-Hirn-Trauma vs. Wirbelkörperfraktur vs. anderes) die Austrittsdestination beeinflussten.

Alle statistischen Auswertungen wurden mit der Statistiksoftware SAS Version 9,4 (SAS Institute, Inc., Cary, North Carolina, USA) vorgenommen. Die wiedergegebenen *p*-Werte sind zweiseitig, mit einem Signifikanzniveau von α = 0,05.

## Ergebnisse

Zwischen Januar 2016 und Juli 2017 wurden insgesamt 201 Patienten mit einer sturzbedingten Verletzung in die geriatrische Abteilung transferiert. Von 173 lag ein schriftliches Einverständnis zur Weiterverwendung ihrer Daten vor. Neun weitere Patienten wurden aufgrund fehlender Austrittsdaten von den Analysen ausgeschlossen: Drei Patienten verstarben während ihres Aufenthalts, bei 6 Patienten konnte kein Austrittsassessment durchgeführt werden. Somit konnten wir 164 Patienten mit einem mittleren Alter 83,3 Jahren, davon 76 % Frauen, in die Analysen einschließen.

Die Charakteristika der eingeschlossenen Patienten zeigt Tab. 1. Die Hauptdiagnosen waren bei 49 (30 %) der Patienten eine Hüft- oder Beckenringfraktur, bei 32 (19,5 %) ein Schädel-Hirn-Trauma, bei 28 (17,1 %) eine Wirkbelkörperfraktur, und bei 55 (33,5 %) lag eine andere traumatologische Hauptdiagnose vor. 146 (89,6 %) der Patienten lebten vor der Hospitalisierung selbstständig zu Hause. Die Mehrheit der Patienten (62 %) hatte einen MMST im normalen Bereich. Zwei Drittel aller Patienten hatten einen MNA-Wert im Bereich der Mangelernährung oder ein erhöhtes Risiko für Mangelernährung. Die Analysen zeigten weiter, dass eine Vielzahl der Patienten mehrere Nebendiagnosen aufwies: 91 % aller Patienten hatten mehr als 5 Nebendiagnosen, 40 % mehr als 10 Nebendiagnosen. Die 5 häufigsten Komorbiditäten waren (in absteigender Reihenfolge): Osteoporose, arterielle Hypertonie, Mangelernährung, Vorhofflimmern, chronische Niereninsuffizienz Stadium 3.

**Table 1:** 

	*n*	Total	Frauen	Männer	*p*-value
*Alter in Jahren; Mittelwert (SA)*	164	83,3 (6,6)	83,7 (6,6)	82,1 (6,3)	0,21
*Wohnsituation: zu Hause lebend, n (%)*	163	146 (89,6)	111 (89,5)	35 (89,7)	0,97
*BMI in kg/m*^*2*^*; Mittelwert (SA)*	155	24,8 (5,0)	24,6 (5,1)	25,5 (4,4)	0,32
*MNA; Mittelwert (SA)*	158	21,9 (3,8)	21,8 (3,8)	22,5 (3,5)	0,31
Normaler Ernährungszustand, *n* (%)	–	53 (33,5)	39 (32,5)	14 (36,8)	0,35
Risiko für Mangelernährung, *n* (%)	–	92 (58,2)	69 (57,5)	23 (60,5)	–
Mangelernährung, *n* (%)	–	13 (8,2)	12 (10,0)	1 (2,6)	–
*MMST; Mittelwert (SA)*	157	23,7 (5,4)	23,8 (5,2)	23,1 (6,1)	0,44
Normale Kognition, *n* (%)	–	98 (62,4)	77 (63,6)	21 (58,3)	0,25
Milde Kognitionseinschränkung, *n* (%)	–	34 (21,7)	26 (21,5)	8 (22,2)	–
Moderate Kognitionseinschränkung, *n* (%)	–	20 (12,7)	16 (13,2)	4 (11,1)	–
Schwere Kognitionseinschränkung, *n* (%)	–	5 (3,2)	2 (1,7)	3 (8,3)	–
*Anzahl der Komorbiditäten; Mittelwert (SA)*	163	10,0 (3,6)	9,8 (3,5)	10,6 (3,7)	0,21
0–5 Komorbiditäten, *n* (%)	–	14 (8,6)	11 (8,9)	3 (7,7)	0,29
6–10 Komorbiditäten, *n* (%)	–	83 (50,9)	67 (54,0)	16 (41,0)	–
>10 Komorbiditäten, *n* (%)	–	66 (40,5)	46 (37,1)	20 (51,3)	–
*Hauptdiagnose*	163	–	–	–	–
Hüftfraktur^a^, *n* (%)	–	49 (29.9)	39 (31,2)	10 (25,6)	0,43
Schädel-Hirn-Trauma^b^, *n* (%)	–	32 (19,5)	21 (16,8)	11 (28,2)	–
Wirbelkörperfraktur, *n* (%)	–	28 (17.1)	21 (16,8)	7 (17,9)	–
Andere, *n* (%)	–	55 (33,5)	44 (35,2)	11 (28,2)	–
*Vollbelastung während Akutspital möglich, n (%)*	163	156 (95,7 %)	120 (96,0 %)	36 (94,7 %)	0,66
*Delir, n (%)*	164	27 (16,5 %)	16 (12,8 %)	11 (28,2 %)	0,044

Diese Tabelle enthält nicht adjustierte Durchschnittswerte oder Prozentangaben. Prozentzahlen auf eine Nachkommastelle gerundet

*SA* Standardabweichung, *MNA* Mini Nutritional Assessment, *MMST* Mini Mental Status Test

^a^Inklusive Femur-, Schenkelhals- und Beckenringfrakturen

^b^Inklusive Hirnblutung

### Verbesserungen von Mobilität und Barthel-Index

Die Aufenthaltsdauer auf der geriatrischen Abteilung lag im Mittel bei 12 Tagen (Median, Interquartilsabstand 8 bis 13 Tage).

In Tab. 2 sind die Resultate der Veränderung der Mobilität und der Selbstständigkeit, nach Geschlecht stratifiziert, dargestellt. Im Rahmen der GFK verbesserte sich der SPPB-Score um 1,8 Punkte (95 %-Vertrauensintervall 1,4–2,1, *p*-Wert <0,001). Männer steigerten sich von initial 2,5 auf 4,2 (*p* < 0,001) und Frauen von 2,2 auf 4,0 (*p* < 0,001). Die statistisch signifikante Verbesserung des SPPB-Scores blieb auch im adjustierten Regressionsmodell bestehen (*p* = 0,031).

**Table 2:** 

	Frauen				Männer				
Mobilitätsparameter	*n*	Beginn	Abschluss	*p*-Wert	*n*	Beginn	Abschluss	*p*-Wert	
*SPPB, Punkte*	111	2,2 (2,6)	4,0 (2,6)	<0,001	35	2,5 (2,8)	4,2 (3,4)	<0,001	
*4-m‑Gehtest der SPPB*									
Nicht fähig zu gehen, *n* (%)	–	49 (44,5 %)	12 (10,9 %)	0,005	–	14 (38,9 %)	8 (22,2 %)	0,036	
Ganggeschwindigkeit^a^, m/s	59	0,4 (0,2)	0,51 (0,19)	<0,001	20	0,45 (0,16)	0,52 (0,2)	0,016	
*Barthel-Index, Punkte*	107	55 (22)	67 (21)	<0,001	27	51 (23)	66 (23)	<0,001	

Gezeigt sind Mittelwerte (Standardabweichungen), sofern nicht anders angegeben. Unterschiede zwischen Eintritts- und Austrittswerten wurde mittels Student’s *t*-Test (kontinuierliche Variablen) und McNemar’s Test (Gehfähigkeit) geprüft

*SPPB* Short Physical Performance Battery

^a^ Für diejenigen, die zu Beginn und vor Entlassung den Gehtest absolvieren konnten

Zu Beginn waren 63 (43,2 %) Patienten nicht in der Lage, den 4‑m-Gehtest durchzuführen; bei Abschluss der GFK reduzierte sich diese Zahl auf 20 (13,7 %) Patienten (*p* < 0,001). Die 79 Patienten, die den 4‑m-Gehtest sowohl bei Ein- als auch bei Austritt absolvierten, zeigten eine statistisch signifikante Verbesserung der Gehgeschwindigkeit um 0,10 m/s (0,07–0,13, *p* < 0,001). Frauen (*n* = 59) verbesserten sich von 0,40 ± 0,20 auf 0,51 ± 0,19 (*p* < 0,001) und Männer von 0,45 ± 0,16 auf 0,52 ± 0,20 (*p* = 0,016).

Der Barthel-Index verbesserte sich im Rahmen der GFK um 12,9 (10,2–15,6, *p* < 0,001) Punkte. Frauen verbesserten sich von 54,7 ± 21,7 auf 67,1 ± 21,0 (*p* < 0,001) und Männer von 51,5 ± 23,1 auf 66,5 ± 23,2 (*p* < 0,001).

Die Veränderungen in der Mobilität und Selbsthilfefähigkeit war bei den verschiedenen Traumagruppen (Hüft‑/Beckenringfraktur, Schädel-Hirn-Traumata, Wirbelkörperfraktur und andere; **Zusatzmaterial online: Tabelle s1 und Tabelle s2**) vergleichbar.

### Short Physical Performance Battery und Barthel-Index als prognostische Tools

Von den 146 Patienten, welche vor Spitaleintritt zu Hause lebten, konnten 104 (71,2 %) wieder zurück nach Hause (61 (43,0 %)) oder als Überbrückung in eine zusätzliche spitalexterne Rehabilitation (43 (30,3 %)) entlassen werden, 38 Patienten (26,0 %) wurden in einer Akut- und Übergangspflege nachbehandelt. Vier (2,7 %) Patienten wurden in andere Kliniken verlegt und nicht in die Analyse eigeschlossen.

**Figure 1:**
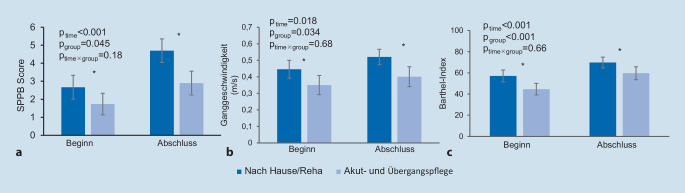

Den geschlechts- und altersadjustierten SPPB-Score, Ganggeschwindigkeit und Barthel-Index zu Beginn und Abschluss der GFK, stratifiziert nach Austrittsdestination, zeigt Abb. 1.

Mit jedem zusätzlichen SPPB-Punkt zu Beginn und jedem zusätzlichen Punktgewinn über die GFK erhöhte sich die Wahrscheinlichkeit, nach Hause oder in eine Reha entlassen zu werden. um 39 % („odds ratio“ 1,39 (1,11–1,73); *p* = 0,004), respektive 33 % (OR 1,33 (1,06–1,68); *p* = 0,01).

Patienten, die nach Hause oder in eine Reha austraten, zeigten eine schnellere Ganggeschwindigkeit zu Beginn und vor Spitalentlassung, verglichen mit Patienten, die in eine Pflegeinstitution verlegt wurden (*p* = 0,03). Patienten mit Weiterbehandlung in einer Pflegeinstitution hatten ein 3,11-fach (1,12–8,65; *p* = 0,03) höheres Risiko, zum Zeitpunkt des Austritts noch nicht gehen zu können, verglichen mit den Patienten, welche zurück nach Hause oder in eine Reha entlassen wurden.

Der Barthel-Index beider Patientengruppen steigerte sich im Verlauf der GFK (p_time_ < 0,001) mit höheren Werten zu Beginn und Entlassung für die Patienten, welche zurück nach Hause oder in eine Reha entlassen wurden, verglichen mit denjenigen, welche in eine Pflegeinstitution austraten (*p*_group_ < 0,001): 57,1 (51,5–62,7) vs. 44,6 (39,2–50,0) zu Beginn (*p* < 0,001) und 69,7 (64,6–74,8) vs. 59,6 (53,5–65,8) vor Entlassung (*p* = 0,006). Mit jeden zusätzlichen 10 Punkten im Barthel-Index zu Beginn erhöhte sich die Chance, nach Hause oder in eine Reha entlassen zu werden um 42 % (OR 1,42 (1,13–1,78); *p* = 0,003). Weder die Anzahl der Komorbiditäten noch die Art des Traumas hatten dabei einen Einfluss auf die Austrittsdestination.

## Diskussion

Im Rahmen der GFK beobachteten wir eine signifikante Verbesserung von Mobilität, Ganggeschwindigkeit und Selbsthilfefähigkeit bei Alterstraumatologiepatienten. Drei von 4 vor ihrem Unfall zu Hause lebende Patienten konnten wieder nach Hause oder als Überbrückung in eine stationäre Rehabilitation austreten.

Unser Patientenkollektiv spiegelt typische alterstraumatologische Patienten mit signifikanter Mobilitätseinschränkung, hoher Inzidenz an Komorbiditäten sowie Risiko für Malnutrition und Kognitionsstörungen wider und entspricht der publizierten Literatur [14–16].

Kwetkat et al. zeigten in einer Analyse von mehr als 110.000 Datensätzen ≥70-jähriger Patienten, dass sich durch die GFK die Mobilität und Selbsthilfefähigkeit signifikant verbesserte und mehr als 80 % der Patienten wieder nach Hause austreten konnten [17]. Eine kleinere Beobachtungsstudie von 105 Patienten mit proximaler Hüftfraktur zeigte ähnliche Verbesserungen der Selbsthilfefähigkeit und Rückkehr nach Hause im Rahmen der GFK [18].

Die von uns beobachtete Verbesserung der Ganggeschwindigkeit von 0,10 m/s und des Barthel-Index um 12,9 Punkte entspricht den Ergebnissen von Kwetkat und Buecking [17, 18]. In unserer Studie zeigte sich, vgl. zu Kwetkat und Buecking, jedoch eine niedrigere Zahl der Rückkehrer nach Hause von 73 %. Möglicherweise ist das durch die unterschiedlichen Patientenpopulationen bedingt.

Sowohl die Ganggeschwindigkeit (von 0,41 auf 0,51 m/s) als auch die Anzahl gehfähiger Patienten (von 57 auf 86,3 %) erhöhte sich während der GFK signifikant. Eine Verbesserung der Ganggeschwindigkeit von 0,1 m/s kann als klinisch signifikante Verbesserung bei alterstraumatologischen Patienten gewertet werden und ist mit anderen Studien älterer Patienten mit Hüftfraktur vergleichbar [19–21].

Die Zunahme des SPPB-Scores von 1,8 Punkten stellt ebenfalls eine klinisch signifikante Verbesserung dar; Perera beschreibt eine Zunahme von 1,0 Punkten als substanziellen Wechsel [22].

Unsere Daten zeigten, dass sowohl SPPB als auch Barthel-Index als prognostische Tools bezüglich Austrittsdestination verwendet werden können: Mit jedem zusätzlichen SPPB-Punkt bei Eintritt und jedem zusätzlichen Punktgewinn über die GFK erhöhte sich die Chance, nach Hause oder in eine Reha entlassen zu werden, um 39 %. Mit jeden zusätzlichen 10 Punkten im Barthel-Index bei Eintritt erhöhte sich die Chance, nach Hause oder in eine Reha entlassen zu werden, um 42 %.

Die Stärken dieser Studie sind, dass wir über 80 % aller alterstraumatologischer Patienten mit der typischen geriatrischen Multimorbidität einschließen konnten. Unsere Beobachtungsstudie umfasst das komplette Spektrum der Alterstraumatologie von der Sprunggelenkfraktur über die Hüftfraktur bis zum Schädel-Hirn-Trauma und liefert somit auch Daten abseits der bereits gut dokumentierten Hüftfrakturen. Eine weitere Stärke ist die standardisierte Erfassung der relevanten Mobilitätsparameter und der anderen geriatrischen Scores. Das Fehlen einer Kontrollgruppe lässt offen, inwiefern die verbesserten Parameter Folge der GFK sind, oder einem natürlichen Heilungsverlauf entsprechen. Eine weitere Limitation ist die limitierte Patientenzahl, die zwar ein generelles Bild von Mobilitätsveränderungen zeigt, für eine detailliertere Analyse der Traumasubgruppen aber zu gering ist.

Die von uns beobachteten Verbesserungen von Mobilität, Ganggeschwindigkeit und Selbsthilfefähigkeit und die publizierte Literatur zu akutgeriatrischen Rehabilitation legen nahe, dass die GFK ein vielversprechendes Behandlungskonzept für multimorbide alterstraumatologische Patienten darstellt [7, 23].

## Fazit für die Praxis

- Im Rahmen der GFK beobachteten wir eine signifikante Verbesserung von Mobilität, Ganggeschwindigkeit und Selbsthilfefähigkeit bei multimorbiden Alterstraumatologiepatienten.
- Mit jedem zusätzlichen SPPB-Punkt zu Beginn und jedem zusätzlichen Punktgewinn im Rahmen der GFK erhöhte sich die Chance, nach Hause oder in eine Reha entlassen zu werden, um 39 %.
- Mit jeden zusätzlichen 10 Punkten im Barthel-Index zu Beginn erhöhte sich die Chance, nach Hause oder in eine Reha entlassen zu werden, um 42 %.

## Caption Electronic Supplementary Material




