# Supplementary material for: Acute geriatric treatment of older trauma patients: Influence on mobility, autonomy and postdischarge destination
Source: Z Gerontol Geriatr. 2020 Nov 17;54(8):816–22. [Article in German] doi: 10.1007/s00391-020-01812-4 (PMC8636419; doi:10.1007/s00391-020-01812-4)
Supplement: Supplementary file 1 [file 391_2020_1812_MOESM1_ESM.docx]

| **Tabelle s1:** Mittelwerte und Standardabweichungen der Mobilitätstest zu Beginn und bei Abschluss der geriatrischen frührehabilitativen Komplexbehandlung stratifiziert nach Patientengruppe | | | | |
| --- | --- | --- | --- | --- |
| Mobilitätsparameter | n | Beginn | Abschluss | *p*-Wert |
| **SPPB, Punkte** |  |  |  |  |
| Hüft- & Beckenringfrakturen | 44 | 1 (1,5) | 2,9 (2,4) | <0.0001 |
| Schädelhirntraumata | 31 | 2,9 (2,6) | 4,1 (2,3) | 0.0004 |
| Wirbelkörperfrakturen | 27 | 3,4 (3,4) | 5,1 (3,2) | <0.0001 |
| Andere | 44 | 2,3 (2,7) | 4,5 (2,6) | <0.0001 |
| **4-m-Gehtest der SPPB** |  |  |  |  |
| **Nicht fähig zu gehen, n (%)** |  |  |  |  |
| Hüft- & Beckenringfrakturen |  | 25 (56,8%) | 11 (25,0%) | 0.0005 |
| Schädelhirntraumata |  | 10 (33,3%) | 4 (13,3%) | 0.0339 |
| Wirbelkörperfrakturen |  | 6 (22,2%) | 2 (7,4%) | 0.1573 |
| Andere |  | 22 (48,9%) | 3 (6,7%) | <0.0001 |
| **Ganggeschwindigkeit^a^, m/s** |  |  |  |  |
| Hüft- & Beckenringfrakturen | 18 | 0,27 (0,1) | 0,4 (0,12) | 0.0019 |
| Schädelhirntraumata | 19 | 0,48 (0,14) | 0,58 (0,18) | 0.3402 |
| Wirbelkörperfrakturen | 19 | 0,45 (0,25) | 0,57 (0,23) | 0.0003 |
| Andere | 23 | 0,45 (0,17) | 0,57 (0,17) | 0.0024 |
| **Barthel-Index, Punkte** |  |  |  |  |
| Hüft- & Beckenringfrakturen | 41 | 46 (19) | 56 (21) | 0.0004 |
| Schädelhirntraumata | 26 | 56 (23) | 72 (22) | <0.0001 |
| Wirbelkörperfrakturen | 23 | 60 (21) | 73 (19) | 0.0032 |
| Andere | 44 | 57 (23) | 71 (19) | <0.0001 |
| ^a^für diejenigen, die bei Ein- und Austritt den Gehtest absolvieren konnten. *SPPB:* Short Physical Performance Battery. P-Werte resultieren aus gepaarten t-Tests. Für die Gehfähigkeit wurde ein McNemar-Test verwendet. | | | | |

**Tabelle s2:** Top 10 der Komorbiditäten

| **Komorbidität (ICD-10-Code)** | Anzahl Patienten (n=164) |
| --- | --- |
| Osteoporose (M80, M81) | 93 |
| Arterielle Hypertonie (I10) | 77 |
| Energie- und Eiweißmangelernährung (E43, E44, E44.1, E46) | 77 |
| Vorhofflimmern (I48) | 59 |
| Chronische Nierenkrankheit, Stadium 3 (N18.3) | 40 |
| Chronische ischämische Herzkrankheit (I25) | 39 |
| Herzinsuffizienz (I50) | 38 |
| Atherosklerose (I70) | 35 |
| Hypertensive Herzkrankheit (I11) | 29 |
| Delir (F05) | 27 |
